# Multi-Omics Analysis of the Gut-Brain Axis Elucidates Therapeutic Mechanisms of Guhong Injection in the Treatment of Ischemic Stroke

**DOI:** 10.3390/ijms26041560

**Published:** 2025-02-12

**Authors:** Pingting Mao, Jianhua Hu, Xi Mai, Na Li, Yijing Liao, Lihua Feng, Qinghong Long

**Affiliations:** School of Pharmacy, Nanchang University, Nanchang 330006, China; pingtingmao@sina.com (P.M.); cici0821@163.com (J.H.); lin@ncu.edu.cn (N.L.); yijingliao@ncu.edu.cn (Y.L.); jxncflh@163.com (L.F.); longqinghong2001@163.com (Q.L.)

**Keywords:** Guhong injection, ischemic stroke, gut microbiota, metabolomics, network pharmacology, gut–brain axis

## Abstract

Guhong injection (GH) is a compound preparation widely utilized in the treatment of cerebrovascular diseases. Accumulating evidence indicates that the gut microbiota is implicated in the development of ischemic stroke (IS). However, although the therapeutic potential of GH in IS may be mediated through the gut microbiota, the intricate relationships among the gut–brain axis, biomarkers, and target proteins remain to be completely explained. A rat model of middle cerebral artery occlusion (MCAO) was utilized to investigate the impact of GH on IS. Our 16S rRNA sequence analysis revealed that GH markedly enhanced the α-diversity of the intestinal microbiome and rectified the imbalance of short-chain fatty acids (SCFAs). Metabolomic analysis indicated that GH reversed 45 biomarkers and 6 disordered metabolic pathways in MCAO rats. Among these, the metabolic pathways of arachidonic acid, α-linolenic acid, fructose, and mannose were closely associated with gut microbiota comprising Lactobacillus modulated by GH. Furthermore, IS-related signaling pathways, including inflammation, autophagy, oxidative stress, and apoptosis, were significantly associated with three gut microbial species influenced by GH. The potential efficacy of GH in the context of IS is mediated through multiple pathways, involving the gut microbiota, SCFAs, biomarkers, and target proteins. This process partly relies on the gut–brain axis.

## 1. Introduction

Ischemic stroke (IS), a prevalent neurological disorder, poses a significant threat to human life. Between 1990 and 2019, there was a 70% increase in the global absolute incidence of stroke and an 85% rise in its prevalence [1], which is partially attributable to both population growth and aging. Nevertheless, the rising age-standardized incidence of IS among individuals aged 18 to 50 (increasing by 50% in the past decade) has received significant attention [2]. It is mainly caused by thrombosis resulting from cerebral atherosclerosis, resulting in vascular stenosis and occlusion. The formation of a thrombus reduces cerebral perfusion, causing brain ischemia and hypoxia, ultimately leading to necrosis and the softening of brain tissue [3]. The multifaceted pathological mechanisms of IS involve various factors, including aberrant brain tissue metabolism, oxidative stress, free radical production, inflammatory response, and apoptosis [4]. Research on IS has progressed from a single, brain-centric perspective of IS to a more comprehensive “whole body” approach. Increasing evidence substantiates the function of intestinal microbiota in gut–brain axis signaling after stroke [5]. An examination of the cellular and molecular immune mechanisms active in the gut–brain axis inflammatory pathway has revealed that disrupted gut microflora, altered intestinal microenvironments, and chronic diseases can exacerbate the prognosis of IS [6]. Alterations in the gut–brain axis have been associated with the pathogenic mechanisms of various diseases, including neurodevelopmental disorders, neurodegenerative diseases, psychiatric disorders, and cerebrovascular accidents such as stroke [7]. Recent research has shown a correlation between the gut microbiota and IS by means of the gut–brain axis, which influences the pathogenesis of stroke [8,9]. Consequently, the intestinal microbiome has become a promising therapeutic target for protecting brain function after a stroke. Accumulating evidence indicates that the gut microbiota and short-chain fatty acids (SCFAs) play a pivotal role as key signaling molecules in the interaction between the digestive tract and the central nervous system [10,11,12,13].

Guhong injection (GH) is a compound preparation consisting of safflower (*Carthamus tinctorius* L.) extract and acetylglutamine, serving as a multi-target drug therapy that aligns with the combination drug model in modern medicine [14,15]. It has been authorized by the China Food and Drug Administration for the management of cerebrovascular diseases, including cerebral blood supply deficiency, cerebral thrombosis, cerebral embolism, and convalescent-stage cerebral hemorrhage. GH combines the characteristics of Western medicine and traditional Chinese medicine, exhibiting anticoagulant, antithrombotic, microcirculation-improving, and anti-oxidative stress properties [15,16,17]. Brain metabolomics studies have shown that GH can ameliorate metabolic disorders in ischemic stroke rats by regulating the glutamate–glutamine cycle, glycolysis, nucleic acid metabolism, the TCA cycle, and phospholipid metabolism [18]. GH could attenuate myocardial ischemia–reperfusion injury by activating GSTP and suppressing the ASK1-JNK/p38 pathway [19]. The microbiota–gut–brain axis is intricately linked to the physiology and pathology of both the digestive tract and the central nervous system. However, the therapeutic potential of GH in IS, possibly mediated by the intestinal microbiota, and the relationships between the gut–brain axis, biomarkers, and target proteins have not been elucidated so far. Our research objective is to elucidate the mechanism of action of GH in treating IS using a middle cerebral artery occlusion (MCAO) rat model. This will be achieved through describing a multi-pronged approach that includes 16S rRNA gene sequencing, metabolomics, network pharmacology, and Western blot (WB). This integrated method aims to demonstrate the impacts of GH on the gut microbiota, biomarkers, SCFAs, and target proteins, thereby revealing that these effects may be achieved through the gut–brain axis.

## 2. Results

### 2.1. TTC Staining and Neurological Deficit

**Figure 1:**
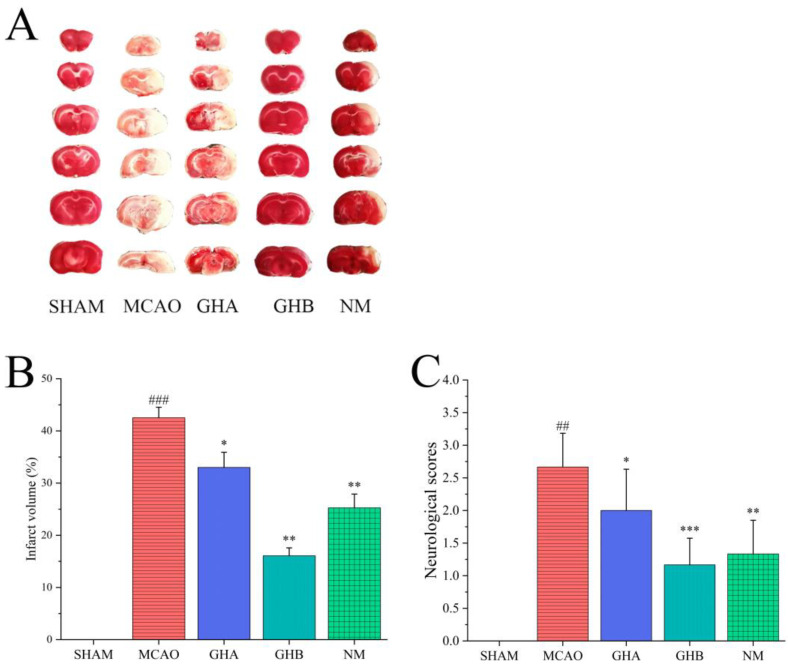
Effect of GH on ischemic brains of rats. (**A**) TTC staining of brains. (**B**) Quantification of infarct volumes. (**C**) Neurobehavioral scores. Data are expressed as mean ± SD (*n* = 3 per group). ^##^ *p* < 0.01, ^###^ *p* < 0.001 compared with SHAM group; * *p* < 0.05, ** *p* < 0.01, *** *p* < 0.001 compared with MCAO group.

The protective effect of GH on IS was assessed through the determination of infarct volume and neurological deficits. TTC staining was performed on brain tissue, where red represented normal tissue and white indicated the ischemic region. Relative to the SHAM group, animals subjected to MCAO exhibited a distinct white area, confirming the successful establishment of the model (Figure 1A). The results revealed that pretreatment with GH and NM significantly improved neurological deficits and infarct volume in the MCAO rats (Figure 1A−C). The infarct volume in the GH and NM (nimodipine injection) groups was significantly reduced compared with that in the MCAO group, suggesting that GH may have the potential to improve blood circulation.

### 2.2. Effects of GH on Gut Microbiota of MCAO Rats

**Figure 2:**
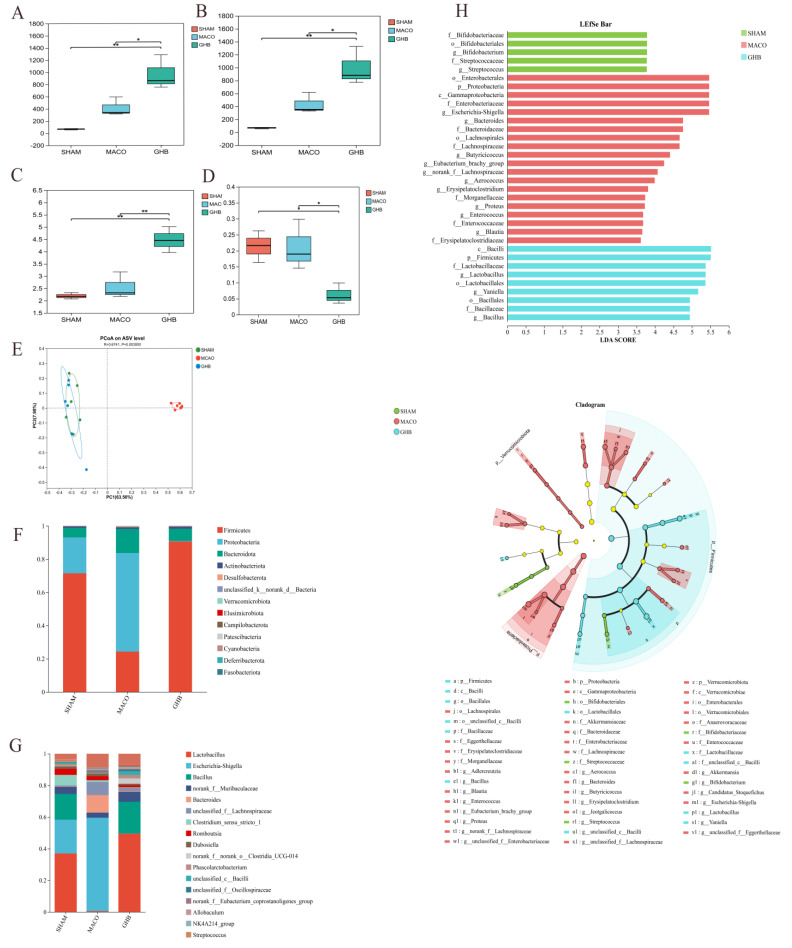
Analysis of the gut microbiota in ischemic stroke rats treated with GH. The α−diversity of the gut microbiota was analyzed using the Chao1 (**A**), Ace (**B**), Shannon (**C**), and Simpson (**D**) indices; (**E**) PCoA analysis of each group. (**F**) Variations in bacterial abundance in different groups at the taxonomic phylum level; (**G**) variations in bacterial abundance in different groups at the taxonomic genus level; (**H**) LEfSe analysis showing the important flora in each group; * *p* < 0.05, and ** *p* < 0.01 compared with the SHAM group.

In this study, high-throughput sequencing produced 2,148,528 optimized sequences, which served to assess the function and composition of gut microbiota. α-Diversity analysis demonstrated that species diversity in the GHB group exceeded that of the MCAO group, as evidenced by the Simpson and Shannon indices. Additionally, species richness in the GHB group, reflected by Chao1 and Ace indices, exceeded that of the MCAO group (Figure 2A−D). Principal coordinate analysis (PCoA) was employed to evaluate the impact of GH on the β diversity of gut microbes, where the proximity on the PCoA plot reflected the similarity in species composition (Figure 2E). The number of ASV corresponding to each phylum and genus level was recorded. Intestinal microbial composition analysis at the phylum level indicated a predominance of Firmicutes in the SHAM group and Proteobacteria in the MCAO group. The results for the GHB group were comparable to those of the SHAM group at the phylum level. LEfSe analysis was performed at the genus level. *Lactobacillus* was predominant in both the SHAM and GHB groups, while *Escherichia-Shigella* was predominant in the MCAO group. Notably, the MCAO group showed an increase in pathogens or conditionally pathogenic bacteria such as *Escherichia-Shigella*, *Enterobacteriaceae*, and *Enterococcus*; however, GH treatment led to an increased abundance of *Lactobacillus* and *Bacillus*, which are considered to be SCFA-producing bacteria (Figure 2F−H).

### 2.3. Effects of GH on SCFAs of MCAO Rats

**Figure 3:**
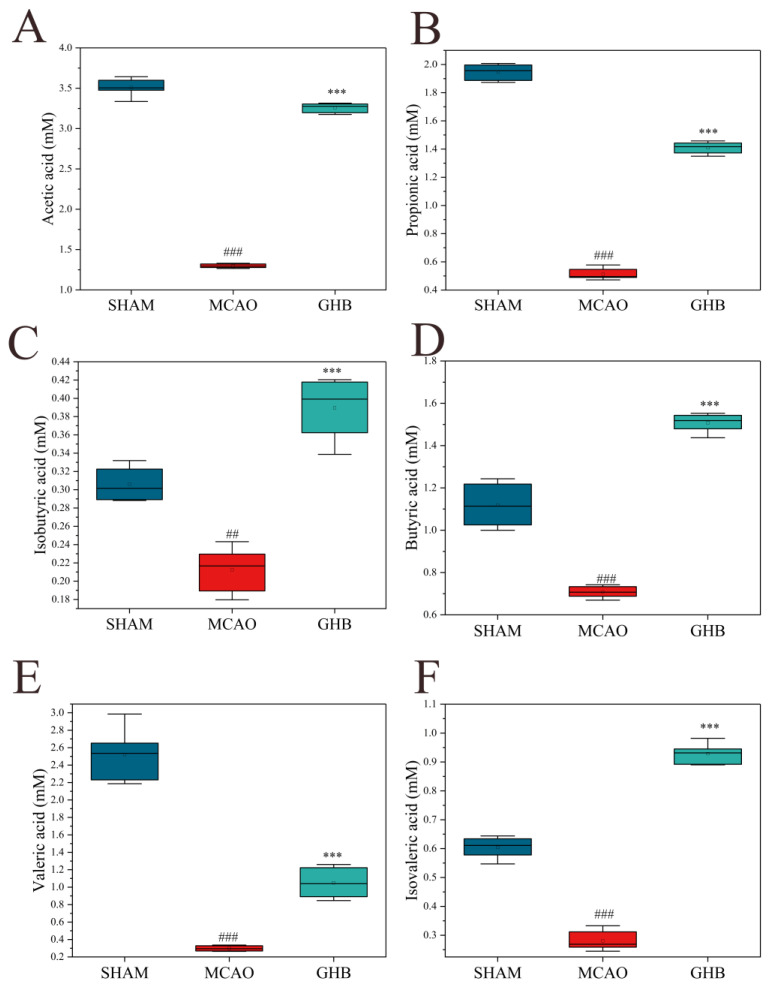
Comparison of SCFAs between groups. (**A**) Acetic acid, (**B**) propionic acid, (**C**) isobutyric acid, (**D**) butyric acid, (**E**) valeric acid, and (**F**) isovaleric acid. Data are expressed as mean ± SD and were analyzed by ANOVA. ## *p* < 0.01; ### *p* < 0.001 compared with SHAM group. *** *p* < 0.001 compared with MCAO group.

The gut microbiota, along with its metabolites, particularly SCFAs, plays a crucial role in preserving the equilibrium of the intestinal microecosystem and enhancing the integrity of the mucosal barrier. Consequently, a quantitative assessment of SCFAs was performed (Table S1). In the MCAO group, we observed that the levels of acetic, propionic, isobutyric, butyric, isovaleric, and valeric acids were significantly lower than those in the SHAM group (Figure 3). In the GHB group, the amounts of acetic, propionic, isobutyric, butyric, isovaleric, and valeric acids were notably elevated compared with the SHAM group (Figure 3).

### 2.4. Metabolomics Analysis

#### 2.4.1. Multivariate Data Analysis

**Figure 4:**
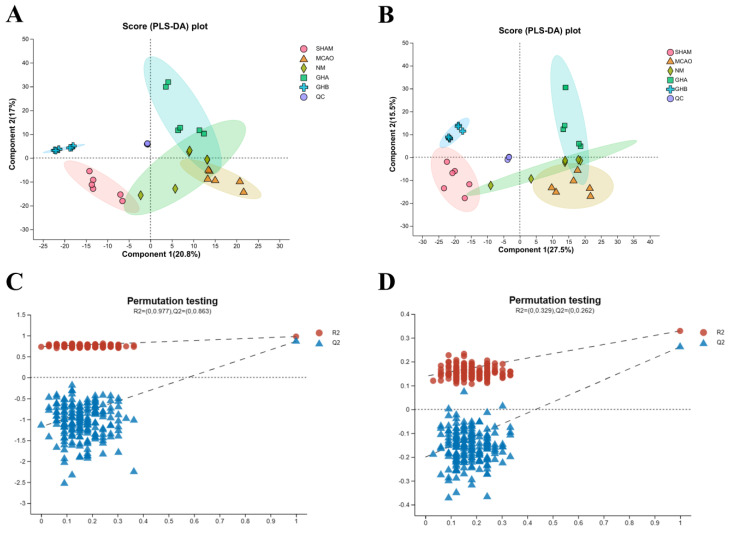
Serum multivariate statistical analysis. PLS-DA score plots for the five groups, including the ESI^+^ (**A**) and ESI^−^ (**B**) ion modes; the results of the permutation test for the five groups, including the ESI^+^ (**C**) and ESI^−^ (**D**) ion modes.

LC-MS was utilized to identify potential metabolites and systematically elucidate the therapeutic mechanism of GH in IS. The total ionic chromatograms (TICs) of serum samples, obtained from negative and positive ion modes, are shown in Figure S1. To evaluate the overall metabolomic changes in IS rats following GH treatment, PCA and PLS-DA models were employed to investigate the trends in metabolic profile alterations among the SHAM, MCAO, GHA, GHB, and NM groups. Our unsupervised PCA of the SHAM, MCAO, GHA, GHB, and NM groups revealed distinct differences in metabolic patterns and natural clustering trends among the groups (Figure S2). The supervised PLS-DA score plot demonstrated significant separation between the groups, indicating successful establishment of the MCAO-induced IS model (Figure 4A,B). Through validation testing, carried out via a permutation test, we ascertained that the PLS-DA model was not overfitted (Figure 4C,D).

To investigate potential biomarkers induced by MCAO and GHB, the raw data of the MCAO group were compared against those of the SHAM and GHB groups using OPLS-DA. The OPLS-DA score plots of serum extracts showed complete separation between the GHB and SHAM groups and the MCAO group, demonstrating Q^2^ > 0.5, while the differences in R^2^Y and Q^2^ were below 0.3. This indicates a pronounced metabolic disorder caused by MCAO, which could be further improved through GHB treatment (Figure S3A,B,E,F). A permutation test was conducted to assess the validity of the OPLS-DA model (Figure S3C,D,G,H). Metabolites with VIP ≥ 1 in the S-plot were considered to significantly contribute to the interference of IS (Figure S3I,J).

#### 2.4.2. Identification of Potential Biomarkers

Potential biomarkers associated with MCAO rats were identified based on a threshold of *p* < 0.05 and VIP > 1.0 for differential metabolites. Consequently, a total of 52 metabolites were filtrated in serum samples between the SHAM and MCAO groups (Table S2). Among these metabolites, 14 were significantly increased, whereas 38 showed a notable reduction in the MCAO group relative to the SHAM group. Notably, administration of GH reversed the variations in 45 metabolites induced by IS (11 downregulated and 34 upregulated). Compared with the MCAO group, the metabolites including PA(8:0/13:0), PC(14:1/20:0), LysoPC(16:1/0:0), PC(16:0/20:2), LysoPC(17:0/0:0), LysoPC(18:1/0:0), LysoPC(P-18:1/0:0), PC(18:2/16:0), LysoPC(18:3/0:0), LysoPC(20:1/0:0), LysoPC(20:5/0:0), 14,15-EET, 19-HETE, L-β-Lysine, and 1-methylhistidine were significantly upregulated in the GH group. Conversely, the metabolites including PC(20:4/18:0), PC(18:3/22:6), PGE2, 4-guanidinebutyric acid, citrulline, creatine, L-dopa, proline, D-sorbitol, mannitol, 2-phenylethanol glucuronide, galactitol, and gentisate aldehyde were downregulated. The Receiver Operating Characteristic (ROC) curve analysis provided insights into the differential metabolites for IS prediction (Figure S4). The results indicated that these metabolites possess strong diagnostic capabilities, with area under the curve (AUC) values ranging from 0.78 to 1.00. Notably, 39 metabolites exhibited excellent diagnostic performance, as evidenced by AUC > 0.90 (Table S3). These findings suggest that these biomarkers hold potential for the clinical diagnosis of IS. However, further human studies are warranted to elucidate their relationships and validate their clinical utility [20,21].

#### 2.4.3. Metabolic Pathways of Potential Biomarkers

**Figure 5:**
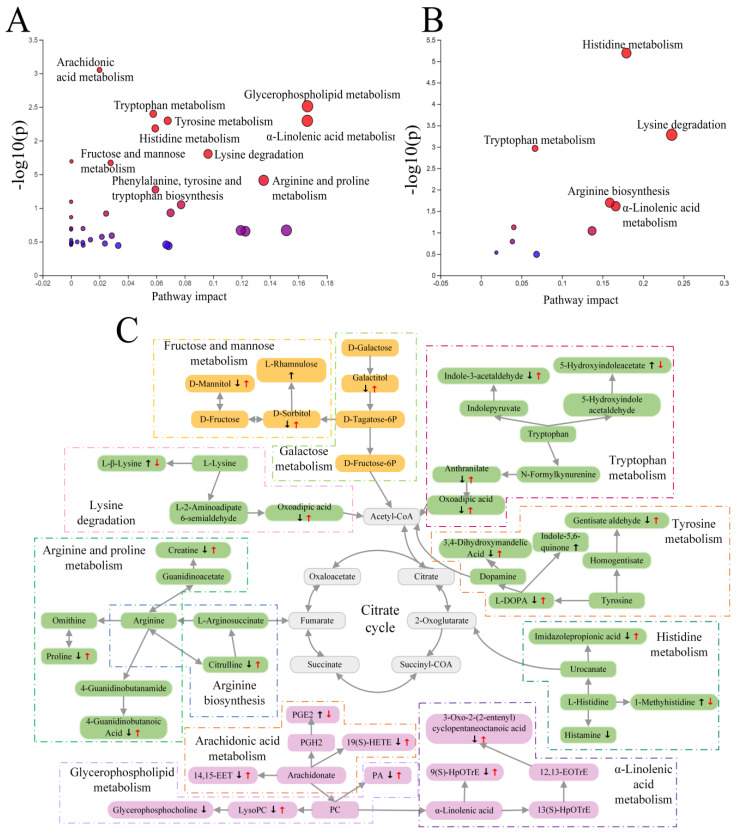
Summary of the pathway analysis of potential biomarkers of ischemic stroke. (**A**) MCAO vs. SHAM; (**B**) GHB vs. SHAM; (**C**) potential metabolic pathways regulated in MCAO rats after treatment with GH. The black arrow indicates upregulation or downregulation in the MCAO group compared to the SHAM group, while the red arrow indicates upregulation or downregulation in GH group compared to MCAO group.

The 52 biomarkers were analyzed for functional enrichment using MetaboAnalyst (Figure 5A). It was observed that the differential metabolites mainly affected 38 metabolic pathways. Among these pathways, 10 were significantly enriched (*p*  <  0.05, impact > 0) (Table S4), including arachidonic acid (ACA) metabolism; glycerophospholipid metabolism; tyrosine metabolism; tryptophan metabolism; α-linolenic acid metabolism; histidine metabolism; arginine and proline metabolism; lysine degradation; fructose and mannose metabolism; and phenylalanine, tyrosine, and tryptophan biosynthesis.

#### 2.4.4. Effects of GH on Metabolic Pathways of MCAO Rats

Comparison between Figure 5A and 5B revealed that the intervention with GH in MCAO rats might have aided the treatment of IS by restoring arachidonic acid metabolism, glycerophospholipid metabolism, arginine and proline metabolism, tyrosine metabolism, fructose and mannose metabolism, and phenylalanine, tyrosine, and tryptophan biosynthesis to normal levels. Using the KEGG database, we investigated the metabolic pathways to create a probable metabolic pathway grid (Figure 5C).

### 2.5. PPI and Disease–Pathway–Target–Drug Network Construction

**Figure 6:**
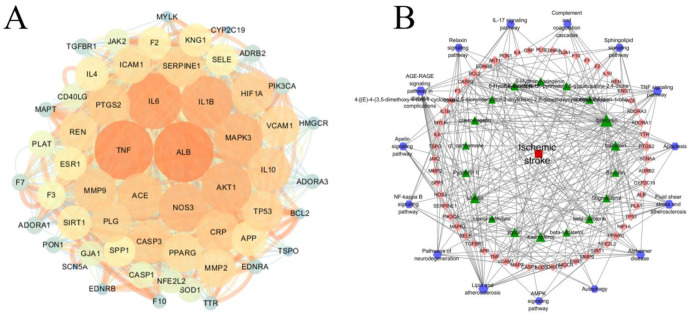
PPI network of GH treatment for ischemic stroke (**A**); drug–target–pathway–disease interaction network of GH (**B**). The green triangles represent the prototype components, the orange-red diamonds represent the targets, the purple hexagons represent the pathways, and the red square represents the disease.

The potential targets of GH were identified using TCMSP and Swiss Target Prediction, leading to the discovery of 528 targets. Additionally, 213 targets related to IS were identified through the Drugbank, OMIM, and Genecard databases (score ≥ 10.0). In total, 56 overlapping targets were found between GH and IS, which were then imported into the String database. The result was imported into cytoscape software for the visual analysis of the PPI network, which consisted of 1422 edges and 56 nodes (Figure 6A). The targets of ALB, TNF, IL6, IL1B, AKT1, NOS3, MAPK3, ACE, MMP9, and PTGS2 were the top 10. The 56 targets were imported into the Metascape database to acquire KEGG and GO terms. The KEGG analysis demonstrated that the treatment of IS with GH possibly involves apoptosis, the NF-κB signaling pathway, the sphingolipid signaling pathway, and autophagy in animals. Based on these results, a drugs–pathways–targets–diseases network was established (Figure 6B).

### 2.6. Effects of GH on Inflammatory Cytokines and Anti-Oxidative Indices in Serum of MCAO Rats

**Figure 7:**
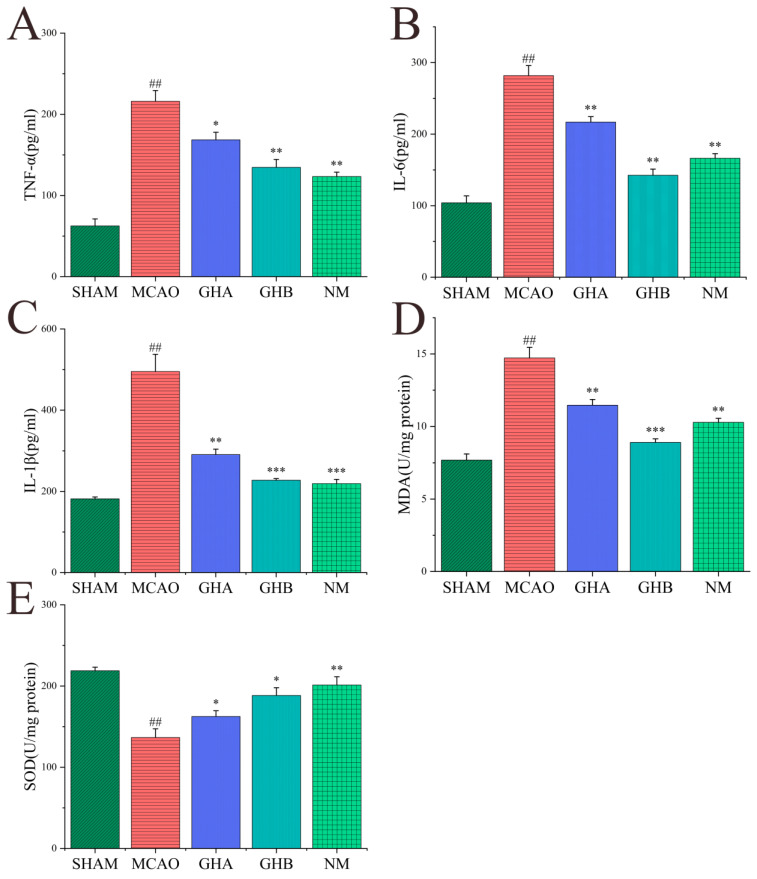
Effects of GH on TNF-α (**A**), IL-6 (**B**), IL-1β (**C**), MDA (**D**), and SOD (**E**) in the serum of MCAO rats. Data are expressed as mean ± SD and were analyzed by ANOVA. ## *p* < 0.01 compared with the SHAM group; * *p* < 0.05, ** *p* < 0.01, and *** *p* < 0.001 compared with the MCAO group.

To evaluate the anti-oxidative and anti-inflammatory effects of GH, serum levels of TNF-α, IL-6, IL-1β, SOD, and MDA were analyzed through ELISA. In comparison to the SHAM group, the levels of MDA, TNF-α, IL-1β, and IL-6 were significantly elevated in the MCAO group. The results indicated a reduction in MDA, TNF-α, IL-1β, and IL-6 levels in the GH and NM groups relative to the MCAO group (Figure 7A–D). Additionally, GH and NM treatment led to the elevation of SOD levels in IS rats (Figure 7E).

### 2.7. The Effects of GH on Inflammatory Responses in the Brains of MCAO Rats

**Figure 8:**
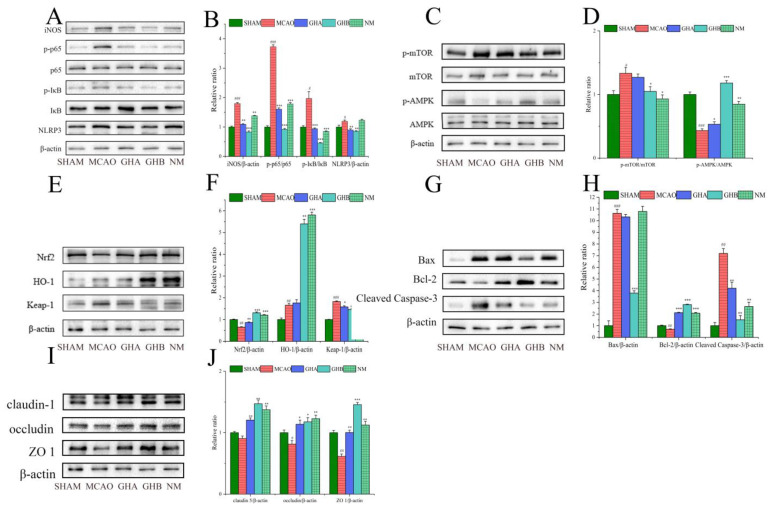
Effects of GH on protein levels associated with various signaling pathways in ischemic stroke rats. (**A**) The protein level of iNOS, p-p65, p65, p- IκBα, IκBα, and NLRP3; (**B**) relative protein expression of iNOS/β-actin, p-p65/p65, p- IκBα/IκBα, and NLRP3/β-actin. (**C**) The protein levels of p-AMPK, AMPK, p-mTOR, and mTOR; (**D**) relative protein expression of p-AMPK/AMPK and p-mTOR/mTOR. (**E**) The protein levels of HO-1, Keap-1, and Nrf2; (**F**) relative protein expression of HO-1/β-actin, Keap-1/β-actin, and Nrf2/β-actin. (**G**) The protein levels of Bax, Bcl-2, and Cleaved Caspase-3; (**H**) relative protein expression of Bax/β-actin, Bcl-2/β-actin, and Cleaved Caspase-3/β-actin. (**I**) The protein levels of claudin-1, occluding, and ZO-1; (**J**) relative protein expression of claudin-1/β-actin, occludin/β-actin, and ZO-1/β-actin. # *p* < 0.05, ## *p* < 0.01, and ### *p* < 0.001 compared with the SHAM group; * *p* < 0.05, ** *p* < 0.01, and *** *p* < 0.001 compared with the MCAO group.

The protein levels of inflammatory response-related biologicals were examined by WB (Figure 8A). The results of the WB analysis indicated that compared to the SHAM group, iNOS, p-IκBα, p-p65, and NLRP3 levels were dramatically increased in the MCAO group. Compared with the MCAO group, the NM group exhibited a downregulation of iNOS, p-p65, and p-IκBα levels, whereas no significant difference was observed in NLRP3 expression (Figure 8B).

### 2.8. The Effects of GH on Autophagy in the Brains of MCAO Rats

Autophagy is essential for the development and occurrence of IS. To investigate the potential regulatory mechanism of GH on autophagy, WB was conducted to evaluate the effects of GH on the expression of mTOR, p-mTOR, AMPK, and p-AMPK (Figure 8C). In the MCAO group, the expression of p-AMPK was reduced, while p-mTOR was increased relative to those in the SHAM group. In the MACO rats treated with GH, the expression of p-mTOR significantly decreased, while that of p-AMPK clearly increased (Figure 8D).

### 2.9. The Effect of GH on Oxidative Stress in the Brains of MCAO Rats

The profiles of HO-1, Nrf2, and Keap-1 in brain tissues are depicted in Figure 8E. In contrast to the SHAM group, the levels of Keap-1 and HO-1 were significantly upregulated, while the Nrf2 content showed a decrease in the MCAO group. Furthermore, compared to the MCAO group, treatment with GH and nimodipine resulted in significant downregulation of Keap-1 expression and a notable increase in the expression levels of HO-1 and Nrf2 (Figure 8F). The therapeutic effect found in the nimodipine group was basically the same as that of the high-dose GHB group.

### 2.10. The Effect of GH on Apoptosis in the Brains of MCAO Rats

To examine the effect of GH on apoptosis in IS rats, WB was utilized to measure the levels of apoptosis-related proteins, including Cleaved Caspase-3, Bax, and Bcl-2, in brain tissue (Figure 8G). Compared to the SHAM group, the levels of Bax and Cleaved Caspase-3 were elevated, while Bcl-2 was reduced in the MCAO group. After intraperitoneal administration of GH and nimodipine in the IS rats, the concentrations of the pro-apoptotic proteins Bax and Cleaved Caspase-3 notably decreased, while the expression of the anti-apoptotic protein Bcl-2 increased (Figure 8H). These findings indicated that the GH treatment groups exhibited a varying degree of regulation in apoptosis.

### 2.11. The Effect of GH on Blood–Brain Barrier (BBB) Integrity in the Brains of MCAO Rats

In order to explore the potential molecular mechanism of GH against BBB disruption in rats with IS, the expression levels of ZO-1, occluding, and claudin-1 in rat brain tissues were assessed using WB (Figure 8I). Compared with the SHAM group, the results indicated a decrease in the levels of ZO-1, occluding, and claudin-1 in the MCAO group. However, treatment with GH and nimodipine injection resulted in a notable enhancement in the expression levels of ZO-1, occludin, and claudin-1 (Figure 8J).

### 2.12. The Interplay Between the Gut Microbiota and Serum Metabolites, SCFAs, and Protein Target Expression After GH Therapy

**Figure 9:**
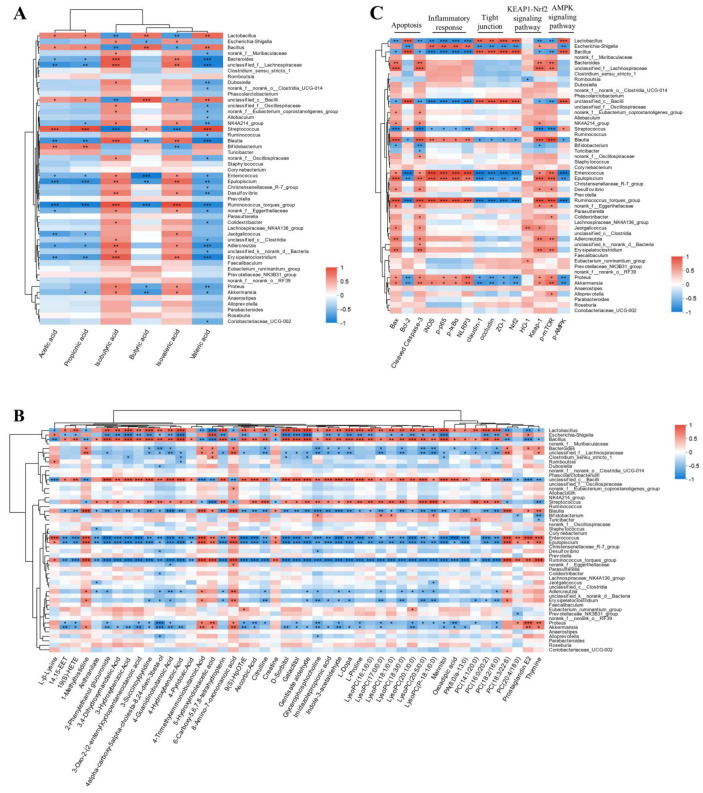
A heat map depicting the association between the gut microbiota and SCFAs (**A**); a heat map depicting the association between gut microbiota and metabolites (**B**); a heat map depicting the association between gut microbiota and signaling pathways (**C**). The vertical coordinates represent different types of intestinal bacteria, while the horizontal coordinates represent the names of SCFAs, metabolites, and protein targets. The color blue denotes a negative correlation, whereas the color red signifies a positive correlation; * *p* < 0.05, ** *p* < 0.01, and *** *p* < 0.001.

Significant variations in the alterations of SCFAs and intestinal microbiota among the SHAM, MCAO, and GHB groups were observed. Spearman correlation analysis was performed to investigate the associations between SCFAs and the gut microbiota within each group. The analysis indicated that 18 bacterial species potentially exert a significant influence on the concentrations of acetic, propionic, isobutyric, butyric, isovaleric, and valeric acids. (*p* < 0.01) (Figure 9A). The gut microbiota most significantly influenced by GH comprised *Lactobacillus*, *Escherichia-Shigella*, and *Bacillus*. Detailed analyses were conducted to elucidate their correlations with SCFAs (Table S5). Spearman correlation analysis showed significant associations between 16 bacterial species and metabolite levels (*p* < 0.01). The most substantial alterations in abundance following GH administration were observed in *Lactobacillus*, *Escherichia-Shigella*, and *Bacillus*. Subsequently, we conducted an analysis to elucidate their correlations with metabolites (Table S6). The observed correlation between the bacteria *Lactobacillus*, *Bacillus*, and *Shigella* and the metabolites phosphatidylcholine (PC), lysophosphatidylcholine (LysoPC), 14,15-EET, L-Dopa, mannitol, and galactitol may suggest a significant role for these microorganisms in the pathophysiology of IS (Figure 9B). The significant associations between the abundance of 16 gut microbial species and signaling pathways, including NF-κB, the NLRP3 inflammasome, AMPK, KEAP1-Nrf2, apoptosis, and tight junctions, were identified by Spearman correlation analysis (Figure 9C) (*p* < 0.01). Furthermore, we performed detailed correlation analyses on the relationship between *Lactobacillus*, *Escherichia-Shigella*, and *Bacillus* and these signaling pathways (Table S7).

## 3. Discussion

In recent years, numerous studies have highlighted the integrative function of the microbiota–gut–brain axis in IS [12,13]. In the present study, analysis of fecal samples using 16S rRNA sequencing revealed that MCAO led to an increase in pathogens or conditionally pathogenic bacteria, including *Escherichia-Shigella*, *Enterobacteriaceae*, and *Enterococcus.* GH treatment significantly enhanced the α-diversity of the intestinal microbiota. The composition of the dominant bacteria in the GH group resembled that of the SHAM group at both the phylum and genus levels, characterized by an increase in the populations of Firmicutes and *Lactobacillus* and a decrease in Proteobacteria and *Escherichia-Shigella*. Previous studies have indicated that *Lactobacillus* administration can confer neuroprotection in IS rat models by inhibiting neuronal apoptosis, diminishing the size of cerebral infarction, attenuating oxidative stress, and ameliorating neurobehavioral deficits [10,22]. Moreover, *Lactobacillus* has been recognized as the primary bacterial strain responsible for elevating SCFA concentrations [23]. SCFAs can exert local effects on the mucosal layer, contributing to the maintenance of intestinal function and barrier integrity [24]. Studies have shown that the levels of acetic, propionic, and butyric acids are lower in stroke patients and older individuals [23,25], and butyric acid mitigates the production of TNF-α and IL-6 induced by LPS [26]. SCFAs have been shown to regulate the gut–brain axis by alleviating epithelial barrier impairment through the facilitation of tight junction formation [24,27,28,29]. Furthermore, they influence the gut–brain axis by governing the differentiation of regulatory Th17 cells, Th1 cells, and T cells [30,31,32]. To elucidate the relationship between GH and the intestinal microbiota more comprehensively, we quantified the concentrations of SCFAs in the intestines of rats. Compared to the SHAM group, the MCAO group showed decreased levels of acetic, propionic, isobutyric, butyric, isovaleric, and valeric acids. After GH intervention, an increase in these SCFAs was observed, suggesting that GH treatment could ameliorate the imbalance of SCFAs induced by MCAO and implying that GH could exert a protective effect on MCAO-induced neuroinflammation, probably due to increased SCFAs. Therefore, the prebiotic properties of GH may facilitate the restoration of the intestinal barrier, attenuate inflammatory responses, and provide neuroprotective effects.

The results of brain metabolomics have shown that GH can ameliorate metabolic disorders by regulating the TCA cycle, glycolysis, nucleic acid metabolism, phospholipid metabolism, and the glutamate–glutamine cycle in IS rats [18], but serum metabolomics-based studies of GH’s effect on IS rats are still needed, and the association with gut microbial dysbiosis in IS is also still unclear. In this study, the results of plasma metabolomics indicated that GH could reverse 45 biomarkers and 6 disordered metabolic pathways in IS rats, including ACA metabolism; tyrosine metabolism; fructose and mannose metabolism; arginine and proline metabolism; glycerophospholipid metabolism; and phenylalanine, tyrosine, and tryptophan biosynthesis. Consistent with brain metabolomics results, the metabolic pathway mainly affected by GH in serum metabolomics is energy metabolism. However, we found that GH affected ACA metabolic pathways and related biomarkers in our serum metabolomics analysis. Substances involved in the metabolism of arachidonic acid, including 14,15-EET and PGE2, are essential in acute ischemic syndromes that impact the coronary and cerebrovascular systems [33]. These substances can inhibit neuronal apoptosis, reduce infarct area, and trigger neuroinflammation following cerebral ischemia [34,35,36]. The correlation analysis of intestinal microbiota and metabolites indicated significant associations between *Lactobacillus*, *Bacillus*, and *Shigella* and metabolites such as phosphatidylcholine (PC), lysophosphatidylcholine (LysoPC), 14,15-EET, L-Dopa, mannitol, and galactitol, underscoring the significant role of these microorganisms in the pathophysiology of IS. Many studies have shown that severe stroke patients have significantly lower phospholipid levels when compared to mild stroke patients and that nerve injury disrupts membrane phospholipid metabolism [37,38]. Dysregulation of phospholipid metabolism, accumulation of lipid peroxides, and energy metabolism impairment may lead to neurodegenerative lesions in ischemia and head injury [37]. Other studies suggest that glycerophospholipid metabolism is involved in MCAO/reperfusion [39,40]. L-dopa, as a precursor of dopamine, plays various roles by binding to postsynaptic dopaminergic receptors in the basal ganglia, affecting neuroplasticity, wakefulness, mood regulation, and motor control [41,42,43]. Guidelines for treating cerebral edema in neurocritical care indicate that mannitol is effective for the early management of cerebral edema or elevated intracranial pressure in acute ischemic stroke patients [44]. Research has indicated that glycerophospholipid metabolism is associated with the gut–brain axis through microglia-mediated neuroinflammation [45]. Additionally, sugar metabolism affects the progression of neurodegenerative diseases and is implicated in the gut–brain axis [46]. It is speculated that the effect of GH on *Lactobacillus*, *Bacillus*, and *Shigella* may be crucial for its anti-inflammatory effects in MCAO rats.

GH has been demonstrated to enhance the prognosis of IS through the regulation of apoptosis, inflammation, and the PI3K/AKT signaling pathway and to attenuate myocardial ischemia–reperfusion injury by activating GSTP and inhibiting the ASK1-JNK/p38 pathway [15,16,19,47]. In the current investigation, GH was found to modulate pro-inflammatory cytokines such as IL-1β, IL-6, and TNF-α, as well as biomarkers of oxidative stress, and regulate target proteins associated with NF-κB, the NLRP3 inflammasome, AMPK, and apoptosis, consistent with previous findings. In addition, three novel findings that differed from previous results were obtained in the current study. First, GH could regulate KEAP1-Nrf2 signaling pathways by increasing the levels of HO-1 and Keap-1, decreasing the level of Nrf2, suggesting that GH could ameliorate IS by mitigating oxidative stress. Nrf2, Keap-1 and HO-1 are target proteins of oxidative stress, which means their effects are induced under oxidative stress and contribute to genomic protection, the exertion of anti-oxidant activity, the scavenging of free radicals, and, ultimately, the provision of neuroprotection [48,49,50]. Second, the results of tight junction signaling pathway-related BBB disruption indicated that GH could increase the expression levels of claudin-1, occludin, and ZO-1. The BBB is a critical component of the neurovascular units, and its disruption significantly increases the risk of mortality in those with early cerebral ischemia. The tight junction (TJ) complex, consisting of transmembrane proteins (occludin, claudin), junction adhesion molecules (JAMs), and cytoplasmic attachment proteins (ZO-1, ZO-2, ZO-3), plays a crucial role in regulating BBB permeability [51]. Thus, these findings indicate that GH treatment may potentially preserve the integrity of the BBB in IS rats. Third, Spearman correlation analysis revealed that p-AMPK, Nrf2, ZO-1, occludin, and claudin-1 exhibited a positive correlation with *Lactobacillus* and a negative correlation with *Shigella*. Conversely, p-mTOR, Keap-1, NLRP3, p-IκBα, p-p65, iNOS, and Bax exhibited negative correlations with *Lactobacillus* and positive correlations with *Shigella*. Finally, the levels of Cleaved Caspase-3 and Bcl-2 demonstrated distinct correlation patterns that require further investigation. Studies have demonstrated that the amelioration of cerebral ischemia–reperfusion injury and Parkinson’s disease, as well as age-related brain injury reduction, are closely associated with the microbiota–gut–brain axis through the reduction in oxidative stress response, inhibition of the NF-κB signaling pathway, and prevention of colonic tight junction protein degradation [52,53,54]. These findings suggest that the impact of GH on oxidative stress and NF-κB signaling pathways may be associated with its regulatory effects on the gut microbiota. Consequently, further research is required to clarify their interrelationships.

**Figure 10:**
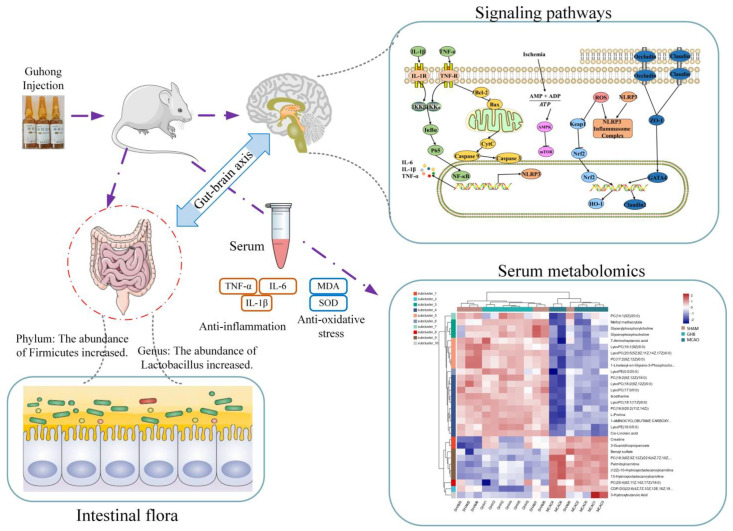
GH exerts its protective effects on ischemic stroke by modulating the gut–brain axis.

Therefore, this study demonstrates that GH can modulate the gut microbiota, SCFAs, biomarkers, and signaling pathways (Figure 10). Nonetheless, this investigation is not devoid of limitations. The study of the impact of GH on the gut microbiota is still novel, and additional studies are needed to clarify how GH plays a role in affecting the gut–brain axis in IS. Changes in the gut microbiome induced by GH can contribute to anti-IS effects in the brain, possibly via humoral and/or other pathways. The specific mechanisms by which GH induces the gut–brain axis remain to be further elucidated. GH can affect the gut microbiome of MCAO rats, but what is its effect on the human gut microbiome? This needs to be further studied so as to provide a scientific basis for the rational application of GH in clinical practice and its clinical action mechanism.

## 4. Materials and Methods

### 4.1. Materials

Guhong injection was obtained from Tonghua Guhong Pharmaceutical Co., Ltd. (Tonghua, China), in which the concentration of aceglutamide was 27–33 mg/mL, and the concentration of hydroxysafflor yellow A was not less than 0.15 mg/mL. Methanol (HPLC grade), acetonitrile (HPLC grade), and isopropanol (HPLC grade) were purchased from Merck Millipore (Molsheim, France). Enzyme-linked immunosorbent assay (ELISA) kits for IL-1β, IL-6, and TNF-α, as well as SOD and MDA kits, were acquired from Wuhan Servicebio Technology Co., Ltd. (Wuhan, China). Monofilament for MCAO was acquired from Beijing Jitai Yuancheng Technology Co., Ltd. (Beijing, China). Antibodies against IκBα, p-IκBα, p65, p-p65, Cleaved Caspase-3, Bcl-2, Bax, HO-1, Nrf2, and Keap-1 were obtained from Abcam (Cambridge, UK). Antibodies against AMPK, p-AMPK, mTOR, p-mTOR, claudin-1, occludin, ZO-1, iNOS, NLRP3, and β-actin were acquired from Cell Signaling Biotechnology (Hertfordshire, UK).

### 4.2. Animals

Male Sprague Dawley rats, weighing 200 ± 20 g, were sourced from SPF (Beijing) Biotechnology Co., Ltd. (China; Certificate No.: SCXK (Jing) 2019-0010). The rats were allowed a one-week adaptation period before the start of the experiment. All rats were kept in a standard animal facility with a 12 h light/dark circular room (with temperature at 25 ± 2 °C and humidity at 60 ± 5%). The rats had free access to tap water and rodent chow. All animal experiments adhered to the Regulations of Experimental Animal Administration and the Guide for the Care and Use of Laboratory Animals, as issued by the State Committee of Science and Technology of Jiangxi Province, China. The study procedure received approval from the Animal Research Ethics Committee of Nanchang University (SYXK(Gan)2021-0004), with animal ethics clearance being granted on 22 December 2021.

### 4.3. Animal Model and Drug Administration

In this study, MCAO was induced following the methods initially outlined by Longa et al. [55]. The rats received anesthesia via an intraperitoneal administration of 1% sodium pentobarbital at a dosage of 3 mL/kg. The subcutaneous tissue and muscle were dissected to expose the common carotid artery (CCA), external carotid artery (ECA), and internal carotid artery (ICA). A monofilament (diameter: 0.24 mm) with a spherical tip was advanced into the ICA to occlude the entry point of the middle carotid artery (MCA). The filament was gently advanced to a depth of about 18–20 mm until slight resistance was felt, and it was left in position for 2 h to induce cerebral ischemia. Subsequently, the filament was slowly retracted to achieve reperfusion. Rats in the sham-operated group underwent identical surgical exposure procedures, but the filament was not inserted.

The rats were randomly assigned to five distinct cohorts: the sham-operated cohort (SHAM group, *n* = 6), MCAO model cohort (MCAO group, *n* = 6), GHA cohort (*n* = 6), GHB cohort (*n* = 6), and positive control drug nimodipine injection cohort (NM, *n* = 6). The rats in the GHA and GHB groups were intraperitoneally injected with 1 mL/kg and 4 mL/kg Guhong injection, respectively (with 1 mL/kg being equivalent to the clinical dose). The NM group rats received a nimodipine injection intraperitoneally at a dosage of 1 mg/kg. The rats in the MCAO and SHAM groups were administered 4 mL/kg of normal saline via intraperitoneal injection daily. The rats were intraperitoneally injected with GH and normal saline 6 h after reperfusion and received continued administration for 7 d.

### 4.4. Assessment of Neurological Impairment

The neurological deficits in rats 24 h post-cerebral ischemia were assessed using the Zea-Longa method. The scoring criteria were as follows: 0 score—no neurological deficits observed; 1 point—inability of the front paw to extend straight when lifted vertically; 2 points—leaning or circling towards the opposite side during walking; 3 points—tilting to the opposite side during walking; and 4 points—inability to walk independently or exhibiting a depressed consciousness.

### 4.5. Sample Collection and TTC Staining

On the 7th day after reperfusion, the rats were anesthetized with 1% sodium pentobarbital (3 mL/kg, i.p.). Blood samples were collected and stored at 4 °C for 2 h. Following this, they underwent centrifugation at 4000 rpm for 10 min at 4 °C, and the resulting serum was kept at −80 °C. The brain was excised and kept at −20 °C for 20 min, before being sliced into 6 pieces (1 mm each). The brain slices were subsequently immersed in a 2% 2,3,5-Triphenyltetrazolium chloride (TTC, Sigma-Aldrich) solution and incubated in the dark at 37 °C for 20 min, with the slices being turned every 5 min. After incubation, the staining solution was removed, and the brain slices were rinsed with PBS to terminate the staining process. Normal brain tissue was visually identified as red, whereas the ischemic regions appeared white.

### 4.6. ELISA

The levels of pro-inflammatory cytokines (IL-1β, IL-6, TNF-α) and indicators associated with oxidative stress (SOD and MDA) were quantified using the corresponding reagent kits.

### 4.7. 16S rRNA Analysis

After collection, the rat feces were promptly kept at −80 °C pending DNA extraction. The V3-V4 segment of the bacterial 16S rDNA gene was chosen for PCR amplification (338 F: 5′-ACTCCTACGGGAGGCAGCAG-3′; 806 R: 5′-GGACTACHVGGGTWTCTAAT-3′). The total volume of the PCR amplification reaction was 20 μL, consisting of the following components: 4 μL of FastPfu Buffer, 10 ng of template DNA, 0.8 μL of the forward primer, 2 μL of dNTPs (2.5 mM each), 0.2 μL of Bovine Serum Albumin (BSA), and 0.8 μL of the reverse primer. The remaining volume was supplemented to 20 μL with ddH_2_O. After quantification, the samples underwent mixing, purification, and recovery procedures. Subsequently, sequencing was conducted by employing the Illumina platform and based on the standardized methods. All valid sequences were classified into amplicon sequence variants (ASVs). The community composition was elucidated using a community bar plot, while species differentials were discerned through linear discriminant analysis effect size (LEfSe).

### 4.8. Quantification of SCFAs

The extraction and quantification methods we used for our analysis of SCFAs were refined based on the protocol established by Zhu et al. [56]. Initially, 50 mg of rat feces was homogenized with 500 µL of PBS and subsequently centrifuged at 4 °C to collect the supernatant. To this supernatant, 200 µL of crotonic acid was added as an internal standard, followed by thorough mixing. The sample was subsequently kept at −20 °C overnight and centrifuged again at 4 °C the next day. The final supernatant, passed through a 0.22 µm filter, was analyzed using gas chromatography (GC-2010 Pro Shimadzu, Kyoto Japan) to determine the concentration of SCFAs in the rat intestinal tract. SCFA contents were quantified using acetic, propionic, butyric, and crotonic acid standards. The measurement was performed using a DB-FFAP column (0.32 mm × 30 m ID) with an injection temperature of 250 °C and a volume of 1 μL. Nitrogen acted as the carrier gas, maintaining a column flow rate of 1 mL/min. The split ratio was adjusted to 8:1, while the scavenging flow rate was kept at 3 mL/min.

### 4.9. LC-MS Metabolomics Analysis

#### 4.9.1. Sample Preparation

The serum sample was retrieved from the refrigerator and allowed to thaw. Subsequently, 100 μL of serum was added to an EP tube, followed by 400 μL of methanol to precipitate the proteins. The mixture was vortexed to ensure thorough mixing and then centrifuged at a speed of 12,000 rpm for 15 min at a temperature of 4 °C. The supernatant (400 μL) was then placed into a sampling vial for UPLC-Q-Exactive MS/MS analysis. The whole procedure was carried out on ice.

#### 4.9.2. LC-MS Conditions

Metabolomics analysis was performed using an ACQUITY UPLC HSS T3 column (100 mm × 2.1 mm i.d., 1.8 µm) integrated with the UHPLC-Q Exactive HF-X system from Thermo Fisher Scientific (USA). The chromatographic separation parameters included a sample injection volume of 3 μL, a flow rate of 0.4 mL/min, and a column temperature set to 40 °C. The mobile phases for the serum samples were composed of solvent A (95% water, 5% acetonitrile, and 0.1% formic acid) and solvent B (a mixture of 47.5% isopropyl alcohol, 47.5% acetonitrile, 5% water, and 0.1% formic acid). The gradient elution was implemented with the following steps: 0–3 min at 10–20% B; 3–4.5 min at 20–35% B; 4.5–5 min at 35–100% B; 5–6.4 min at 100% B; and 6.4–8 min at 0% B.

### 4.10. Multivariate Statistical Analysis and Data Processing

Appropriate statistical analyses, including one-way ANOVA followed by Tukey’s multiple comparison test or Student’s *t*-test, were performed as required to evaluate the data. Raw data from each group were processed using Progenesis QI for LC-MS data processing. A two-dimensional dataset was subsequently loaded into SIMCA-P 14.1 software to carry out orthogonal partial least squares discriminant analysis (OPLS-DA), partial least squares discriminant analysis (PLS-DA), and principal component analysis (PCA). The OPLS-DA, PLS-DA, and PCA models were evaluated using R^2^X, R^2^Y, and Q^2^Y intercepts. Additionally, the OPLS-DA models were validated through cross-validated residual variance testing (CV-ANOVA). The ions of potential biomarkers were filtered using a *p*-value of < 0.05 and variable importance in projection (VIP) values of ≥1.

### 4.11. Network Pharmacological Analysis

The chemical ingredients of GH were ascertained from relevant literature and the Traditional Chinese Medicine Systems Pharmacology (TCMSP) database (https://old.tcmsp-e.com/tcmsp.php, accessed on 9 February 2023). The drug targets were sourced from the TCMSP database and the Swiss Target Prediction Database (http://swisstargetprediction.ch/, accessed on 9 February 2023), while the targets associated with IS were retrieved from the OMIM (http://www.ncbi.nlm.nih.gov/omim, accessed on 9 February 2023) and GeneCards (https://www.genecards.org/, accessed on 9 February 2023) databases.

The filtered targets of GH and IS were assessed for protein–protein interactions (PPIs) using the STRING database (https://stringdb.org/, accessed on 9 February 2023). “Homo sapiens” was selected as the background species, with a confidence score threshold of 0.7, while other parameters were kept at their default settings. Subsequently, a disease–pathway–target–drug network was constructed to investigate the potential mechanisms of GH. The PPI results and the disease–pathway–target–drug data were displayed using Cytoscape 3.7.1 to generate network visualizations.

### 4.12. Western Blot Analysis

The hippocampus and cerebral cortex tissues were collected from the rats’ brains. Brain tissues were lysed using RIPA buffer that included phosphorylation and protease inhibitors. The lysate was then subjected to low-temperature grinding twice (30 s each time), carried out using a grinding tube containing 3 steel balls. After grinding, the lysate was chilled on ice for 30 min and then subjected to centrifugation at 4 °C for 15 min at 12,000× *g*. The supernatant was harvested, and the protein concentration was determined using a BCA protein analysis kit. Equivalent protein sample quantities were applied and separated using 6% to 10% SDS-PAGE. The isolated proteins were then transferred to PVDF membranes, which were subsequently blocked with protein-free rapid blocking buffer for 25 min. The membranes were placed on a shaker and incubated with the primary antibody overnight at 4 °C, followed by a 2 h treatment at room temperature with a peroxidase-labeled secondary antibody. The protein bands were subsequently revealed utilizing an ECL kit.

## 5. Conclusions

In the current study, 52 differential metabolites in a rat model of MCAO were identified, and it was shown that GH could reverse 45 biomarkers and restore 6 dysregulated metabolic pathways associated with IS. Additionally, GH exerted therapeutic effects on IS through the modulation of various signaling pathways, including the NF-κB, NLRP3 inflammasome, AMPK, KEAP1-Nrf2, apoptosis, and tight junction signaling pathways. The 16S rRNA analysis indicated that MCAO increased or conditioned the pathogens in the intestine, and GH treatment increased the α-diversity of the intestinal flora and the abundance of Lactobacillus, subsequently enhancing the production of SCFAs. Spearman correlation analysis indicated significant correlations between 16 bacterial species and metabolite levels, as well as signaling pathways. In summary, GH has been demonstrated to possess potential efficacy in attenuating the inflammatory response, neuronal apoptosis, and BBB damage in IS by modulating multiple gut–brain axis-based pathways.

## Data Availability

Data are available on request from the authors.

## Supplementary Materials

The following supporting information can be downloaded at: https://www.mdpi.com/article/10.3390/ijms26041560/s1.
