# Supplementary material for: Multi-Omics Analysis of the Gut-Brain Axis Elucidates Therapeutic Mechanisms of Guhong Injection in the Treatment of Ischemic Stroke
Source: Int J Mol Sci. 2025 Feb 12;26(4):1560. doi: 10.3390/ijms26041560 (PMC11855775; doi:10.3390/ijms26041560)
Supplement: Supplementary file 1 [file ijms-26-01560-s001.zip › ijms-3452282-supplementary.pdf]

Multi-omics Analysis of the Gut-Brain Axis Elucidates Therapeutic Mechanisms of Guhong Injection in the Treatment of Ischemic Stroke

Pingting Mao [1], Jianhua Hu [1], Xi Mai [1*], Zhiwang Zhou [1], Na Li [1], Yijing Liao [1], Lihua Feng [1], Qinghong Long [1]
[1] School of Pharmacy, Nanchang University, Nanchang 330006, P. R.China

Table S1. The content of SCFAs in each group.. 1

Table S2. Message and Changing Trend of Key Biomarkers in the Rat Serum. 2

Table S3. ROC analysis of differential metabolites. 6

Table S4. Enrichment analysis of potential biomarker pathway of GH in treatment of ischemic stroke. 8

Table S5. Gut microbiota and SCFAs associated with GH. 9

Table S6. Gut microbiota and metabolites associated with GH. 10

Table S7. Gut microbiota and signaling pathways associated with GH........... 13

Figure S1 Total ion chromatograms (TIC) of serum....................................... 14

Figure S2 The PCA score plots of the five group. 15

Figure S3 The OPLS-DA score plots of MCAO vs SHAM............................ 16

Figure S4 ROC analysis of differential metabolites. 18

* Corresponding autor. Fax: 086- 791-86361839
E-mail addresses: maixi123@aliyun.com (X. Mai)

Table S1. The content of SCFAs in each group.

Name	Group		
	SHAM	MCAO	GHB
Acetic acid	3.51±0.11	1.30±0.025 (↓###)	3.26±0.059 (↑***)
Propionic acid	1.95±0.056	0.51±0.041 (↓###)	1.42±0.051 (↑***)
Isobutyric acid	0.31±0.018	0.21±0.054 (↓##)	0.39±0.032 (↑***)
Butyric acid	1.12±0.11	0.71±0.027 (↓###)	1.51±0.045 (↑***)
Isovaleric acid	0.060±0.0037	0.028±0.0034 (↓###)	0.093±0.0035 (↑***)
Valeric acid	0.25±0.029	0.029±0.0030 (↓###)	0.11±0.018 (↑***)

##$p < 0.01$, ###$p < 0.001$ compared with SHAM group; ***$p < 0.001$ compared with MCAO group.

Table S2. Message and Changing Trend of Key Biomarkers in the Rat Serum.

No.	m/z	Name	Retention time (min)	Ion	HMDB	Formula	Trend		
							MCAO/SHAM	GHB/MCAO	GHB/SHAM
1	210.1210	L-β-Lysine	0.5478	ESI^+	HMDB0012114	$C_6H_{14}N_2O_2$	↑*	↓###	↓*
2	734.5683	1,2-Dipalmitoylphosphatidylcholine	7.2729	ESI^+	HMDB0245715	$C_{40}H_{80}NO_8P$	↓*		
3	365.2334	14,15-EET	5.8449	ESI^-	HMDB0242325	$C_{20}H_{32}O_3$	↓*	↑##	↑#
4	365.2335	19(S)-HETE	5.6676	ESI^-	HMDB0011136	$C_{20}H_{32}O_3$	↓*	↑##	↑#
5	170.0923	1-Methylhistidine	0.6987	ESI^+	HMDB0000001	$C_7H_{11}N_3O_2$	↑***	↓###	
6	182.0455	Anthranilate	1.9595	ESI^-	HMDB0001123	$C_7H_7NO_2$	↓***	↑##	
7	297.0982	2-Phenylethanol glucuronide	5.3274	ESI^-	HMDB0010350	$C_{14}H_{18}O_7$	↓***	↑###	
8	205.0116	3,4-Dihydroxymandelic Acid	5.8007	ESI^-	HMDB0001866	$C_8H_8O_5$	↓***	↑###	↑**
9	137.0237	3-Hydroxybenzoic Acid	5.8047	ESI^-	HMDB0002466	$C_7H_6O_3$	↓***	↑###	↑**
10	293.2126	3-Oxo-2-(2-entenyl) cyclopentaneoctanoic acid	5.9468	ESI^-	HMDB0301805	$C_{18}H_{30}O_3$	↓**	↑###	↑***
11	178.0505	3-Succinoylpyridine	3.5181	ESI^-	HMDB0000992	$C_9H_9NO_3$	↓***	↑###	
12	462.2864	4alpha-carboxy-5alpha-cholesta-8,24-dien-3beta-ol	5.6676	ESI^-	HMDB0062385	$C_{28}H_{43}O_{3-}$	↓**	↑###	

No.	m/z	Name	Retention time (min)	Ion	HMDB	Formula	Trend		
							MCAO/SHAM	GHB/MCAO	GHB/SHAM
13	146.0923	4-Guanidinobutanoic Acid	0.8336	ESI^+	HMDB0003464	$C_5H_{11}N_3O_2$	↓***	↑###	
14	137.0237	4-Hydroxybenzoic Acid	5.5459	ESI^-	HMDB0000500	$C_7H_6O_3$	↓***	↑###	↑**
15	184.0602	4-Pyridoxic Acid	1.7930	ESI^+	HMDB0000017	$C_8H_9NO_4$	↓***	↑##	
16	146.1174	4-Trimethylammoniobutanoic Acid	0.7622	ESI^+	HMDB0001161	$C_7H_{15}NO_2$	↑***	↓###	
17	209.0918	5-Hydroxyindoleacetic acid	1.9530	ESI^+	HMDB0000763	$C_{10}H_9NO_3$	↑**	↓##	
18	246.0385	6-Carboxy-5,6,7,8-tetrahydropterin	4.0203	ESI^-	HMDB0060410	$C_7H_9N_5O_3$	↓***	↑###	
19	229.1544	8-Amino-7-oxononanoic acid	0.7464	ESI^+	HMDB0240687	$C_9H_{17}NO_3$	↑**	↓###	
20	309.2075	9(S)-HpOTrE	5.9390	ESI^-	-	$C_{18}H_{30}O_4$	↓***	↑###	↑*
21	174.088	Citrulline	0.5875	ESI^-	HMDB0000904	$C_6H_{13}N_3O_3$	↓**	↑#	
22	132.0767	Creatine	0.6113	ESI^+	HMDB0000064	$C_4H_9N_3O_2$	↓*	↑#	
23	181.0714	D-Sorbitol	0.5875	ESI^-	HMDB0000247	$C_6H_{14}O_6$	↓***	↑###	↑**
24	205.0678	Galactitol	0.7066	ESI^+	HMDB0000107	$C_6H_{14}O_6$	↓**	↑###	↑***
25	180.0653	Gentisate aldehyde	3.5098	ESI^+	HMDB0004062	$C_7H_6O_3$	↓***	↑###	
26	280.0916	Glycerophosphocholine	0.5398	ESI^+	HMDB0008027	$C_8H_{20}NO_6P$	↓**	↑#	
27	112.0871	Histamine	0.5319	ESI^+	HMDB0000870	$C_5H_9N_3$	↓**		↓*

No.	m/z	Name	Retention time (min)	Ion	HMDB	Formula	Trend		
							MCAO/SHAM	GHB/MCAO	GHB/SHAM
28	141.0657	Imidazolepropionic acid	0.8256	ESI^+	HMDB0002271	$C_6H_8N_2O_2$	↓***	↑###	
29	204.0664	Indole-3-acetaldehyde	5.3550	ESI^-	HMDB0001190	$C_{10}H_9NO$	↓***	↑###	↑**
30	148.0392	Indole-5,6-quinone	3.2517	ESI^+	HMDB0006779	$C_8H_5NO_2$	↑*		↑*
31	178.0505	L-Dopa	4.0088	ESI^-	HMDB0000181	$C_9H_{11}NO_4$	↓***	↑###	
32	116.0708	L-Proline	0.6192	ESI^+	HMDB0251528	$C_5H_9NO_2$	↓**	↑###	
33	185.0427	L-Rhamnulose	2.7359	ESI^-	HMDB0010207	$C_6H_{12}O_5$	↑**		↑*
34	171.027	L-Xylulose	2.1353	ESI^-	HMDB0000751	$C_5H_{10}O_5$	↑*		
35	494.3234	LysoPC(16:1/0:0)	5.8895	ESI^+	HMDB0010383	$C_{24}H_{48}NO_7P$	↓**	↑###	
36	510.3546	LysoPC(17:0/0:0)	6.6218	ESI^+	HMDB0012108	$C_{25}H_{52}NO_7P$	↓***	↑###	
37	522.3549	LysoPC(18:1/0:0)	6.5506	ESI^+	HMDB0010385	$C_{26}H_{52}NO_7P$	↓***	↑###	
38	562.3154	LysoPC(18:3/0:0)	5.8047	ESI^-	HMDB0010387	$C_{26}H_{48}NO_7P$	↓***	↑###	
39	550.3857	LysoPC(20:1/0:0)	6.6376	ESI^+	HMDB0010391	$C_{28}H_{56}NO_7P$	↓***	↑###	↑**
40	542.3211	LysoPC(20:5/0:0)	7.2888	ESI^+	HMDB0010397	$C_{28}H_{48}NO_7P$	↓***	↑###	
41	538.3859	LysoPC(P-18:1/0:0)	6.6138	ESI^+	HMDB0010408	$C_{26}H_{52}NO_6P$	↓***	↑#	↑*
42	205.0678	Mannitol	0.5715	ESI^+	HMDB0000765	$C_6H_{14}O_6$	↓**	↑###	↑***

No.	m/z	Name	Retention time (min)	Ion	HMDB	Formula	Trend		
							MCAO/SHAM	GHB/MCAO	GHB/SHAM
43	184.0603	O-Succinyl-L-homoserine	2.1205	ESI^+	HMDB0255868	$C_8H_{13}NO_6$	↓***		↑**
44	178.0708	Oxoadipic acid	0.8097	ESI^+	HMDB0000225	$C_6H_8O_5$	↓**	↑###	↑*
45	512.3343	PA(8:0/a-13:0)	5.9135	ESI^+	HMDB0115680	$C_{24}H_{47}O_8P$	↓***	↑###	
46	782.5676	PC(14:1/20:0)	7.2729	ESI^+	HMDB0007911	$C_{42}H_{82}NO_8P$	↓**	↑#	
47	786.5996	PC(16:0/20:2)	7.2729	ESI^+	HMDB0007979	$C_{44}H_{84}NO_8P$	↓***	↑###	
48	758.5682	PC(18:2/16:0)	6.8677	ESI^+	HMDB0008133	$C_{42}H_{80}NO_8P$	↓***	↑###	
49	828.5502	PC(18:3/22:6)	7.2809	ESI^+	HMDB0008189	$C_{48}H_{78}NO_8P$	↑***	↓###	
50	810.5989	PC(20:4/18:0)	5.9773	ESI^+	HMDB0008464	$C_{46}H_{84}NO_8P$	↑**	↓#	↓*
51	127.0502	Thymine	1.9450	ESI^+	HMDB0000262	$C_5H_6N_2O_2$	↑***	↓###	
52	335.221	Prostaglandin E2	5.8895	ESI^+	HMDB0001220	$C_{20}H_{32}O_5$	↑***	↓###	

*$p < 0.05$, **$p < 0.01$, ***$p < 0.001$, vs SHAM

#$p < 0.05$, ##$p < 0.01$, ###$p < 0.001$, vs MCAO

Table S3. ROC analysis of differential metabolites.

Metabolite	AUC	CI
1,2-Dipalmitoylphosphatidylcholine	0.9444	[0.8156, 1]
1-Methylhistidine	1	[1, 1]
2-Aminobenzoic acid	1	[1, 1]
2-Phenylethanol glucuronide	1	[1, 1]
3,4-Dihydroxymandelic Acid	1	[1, 1]
3-Hydroxybenzoic Acid	1	[1, 1]
3-Oxo-2-(2-entenyl)cyclopentaneoctanoic acid	1	[1, 1]
3-Succinoylpyridine	1	[1, 1]
4alpha-carboxy-5alpha-cholesta-8,24-dien-3beta-ol	0.9444	[0.8156, 1]
4-Guanidinobutanoic Acid	1	[1, 1]
4-Hydroxybenzoic Acid	1	[1, 1]
4-Pyridoxic Acid	1	[1, 1]
4-Trimethylammoniobutanoic Acid	1	[1, 1]
6-Carboxy-5,6,7,8-tetrahydropterin	1	[1, 1]
8-Amino-7-oxononanoic acid	1	[1, 1]
9(S)-HpOTrE	1	[1, 1]
Citrulline	1	[1, 1]
D-Sorbitol	1	[1, 1]
Galactitol	1	[1, 1]
Gentisate aldehyde	1	[1, 1]
Glycerophosphocholine	0.9444	[0.8156, 1]
Histamine	0.9722	[0.8952, 1]
Imidazolepropionic acid	1	[1, 1]
Indole-3-acetaldehyde	1	[1, 1]
L-Dopa	1	[1, 1]
L-Proline	1	[1, 1]

LysoPC(17:0/0:0)	1	[1, 1]
LysoPC(18:1/0:0)	1	[1, 1]
LysoPC(20:1/0:0)	1	[1, 1]
LysoPC(20:5/0:0)	1	[1, 1]
LysoPC(P-18:1(9Z)/0:0)	1	[1, 1]
Mannitol	0.9722	[0.8952, 1]
O-Succinyl-L-homoserine	1	[1, 1]
PA(8:0/a-13:0)	1	[1, 1]
PC(16:0/20:2)	0.9722	[0.8952, 1]
PC(18:2/16:0)	1	[1, 1]
PC(18:3/22:6)	1	[1, 1]
Prostaglandin E2	1	[1, 1]
Thymine	1	[1, 1]

Table S4. Enrichment analysis of potential biomarker pathway of GH in treatment of ischemic stroke.

No.	Name	Match status	*P*	Impact
1	Arachidonic acid metabolism	4/37	0.0008904	0.01997
2	Glycerophospholipid metabolism	4/52	0.003073	0.1664
3	Tryptophan metabolism	4/56	0.003982	0.05779
4	Tyrosine metabolism	4/60	0.005049	0.06797
5	α-Linolenic acid metabolism	3/30	0.005059	0.1662
6	Histidine metabolism	3/33	0.006565	0.05922
7	Lysine degradation	3/46	0.01569	0.09636
8	Fructose and mannose metabolism	3/52	0.02130	0.02779
9	Arginine and proline metabolism	3/67	0.03872	0.1355
10	Phenylalanine, tyrosine and tryptophan biosynthesis	2/33	0.04916	0.05934

Table S5. Gut microbiota and SCFAs associated with GH.

Name	Lactobacillus		Escherichia-Shigella		Bacillus	
	p value	|r|	p value	|r|	p value	|r|
Acetic acid	0.0138	0.5686	0.0700	0.4367	0.0335	0.5026
Propionic acid	0.0151	0.5624	0.1398	0.3620	0.0191	0.5459
Isobutyric acid	0.0027	0.6636	0.0305	0.5104	0.0017	0.6863
Butyric acid	0.0045	0.6367	0.0203	0.5415	0.0017	0.6842
Isovaleric acid	0.0037	0.6471	0.0426	0.4824	0.0138	0.5686
Valeric acid	0.0020	0.6780	0.0520	0.4647	0.0045	0.6367

Table S6. Gut microbiota and metabolites associated with GH.

Name	Lactobacillus		Escherichia-Shigella		Bacillus	
	p value	|r|	p value	|r|	p value	|r|
L-β-Lysine	0.0130	0.5728	0.0156	0.5602	0.0011	0.7049
14,15-EET	0.0126	0.5748	0.0321	0.5062	0.0165	0.5562
19(S)-HETE	0.0030	0.6577	0.0242	0.5283	0.0063	0.6174
1-Methylhistidine	0.0164	0.5568	0.0738	0.4315	0.0066	0.6147
Anthranilate	0.0528	0.4634	0.0868	0.4149	0.0389	0.4902
2-Phenylethanol glucuronide	0.0013	0.6963	0.0020	0.6781	0.0001	0.7781
3,4-Dihydroxymandelic Acid	0.0015	0.6898	0.0018	0.6820	0.0014	0.6940
3-Hydroxybenzoic Acid	0.0003	0.7564	0.0002	0.7728	0.0001	0.7977
3-Oxo-2-(2-entenyl)cyclopentaneoctanoic acid	0.0072	0.6099	0.0099	0.5903	0.0015	0.6904
3-Succinoylpyridine	0.0004	0.7420	0.0018	0.6815	0.0007	0.7214
4alpha-carboxy-5alpha-cholesta-8,24-dien-3beta-ol	0.0067	0.6144	0.0435	0.4805	0.0012	0.7001
4-Guanidinobutanoic Acid	0.0033	0.6533	0.0099	0.5903	0.0011	0.7028
4-Hydroxybenzoic Acid	0.0002	0.7730	0.0001	0.8029	0.0000	0.8349
4-Pyridoxic Acid	0.0590	0.4530	0.1435	0.3589	0.0259	0.5232
4-Trimethylammoniobutanoic Acid	0.0080	0.6037	0.0520	0.4647	0.0033	0.6533
5-Hydroxyindoleacetic acid	0.0000	0.8260	0.0004	0.7437	0.0001	0.7827
6-Carboxy-5,6,7,8-tetrahydropterin	0.0006	0.7294	0.0017	0.6843	0.0005	0.7367
8-Amino-7-oxononanoic acid	0.0123	0.5761	0.0767	0.4276	0.0047	0.6340
9(S)-HpOTrE	0.0029	0.6591	0.0080	0.6038	0.0014	0.6942
Ascorbic Acid	0.0126	0.5748	0.0615	0.4492	0.0050	0.6305

Name	Lactobacillus		Escherichia-Shigella		Bacillus	
	p value	|r|	p value	|r|	p value	|r|
Citrulline	0.0013	0.6987	0.0168	0.5550	0.0014	0.6925
Creatine	0.0290	0.5142	0.0336	0.5023	0.0338	0.5018
D-Sorbitol	0.0002	0.7626	0.0001	0.7770	0.0002	0.7688
Galactitol	0.0003	0.7585	0.0006	0.7272	0.0002	0.7709
Gentisate aldehyde	0.0000	0.8142	0.0000	0.8206	0.0000	0.8101
Glycerophosphocholine	0.0226	0.5335	0.1741	0.3351	0.0380	0.4923
Imidazolepropionic acid	0.0029	0.6594	0.0073	0.6089	0.0013	0.6987
Indole-3-acetaldehyde	0.0028	0.6618	0.0107	0.5854	0.0013	0.6980
L-Dopa	0.0005	0.7358	0.0012	0.7002	0.0004	0.7441
L-Proline	0.0010	0.7090	0.0038	0.6463	0.0004	0.7441
LysoPC(16:1/0:0)	0.0016	0.6883	0.0081	0.6027	0.0011	0.7028
LysoPC(17:0/0:0)	0.0092	0.5950	0.0738	0.4315	0.0139	0.5682
LysoPC(18:1/0:0)	0.0023	0.6701	0.0179	0.5506	0.0052	0.6288
LysoPC(18:3/0:0)	0.0020	0.6784	0.0111	0.5828	0.0018	0.6825
LysoPC(20:1/0:0)	0.0022	0.6739	0.0058	0.6224	0.0005	0.7358
LysoPC(20:5/0:0)	0.0022	0.6742	0.0159	0.5589	0.0012	0.7021
LysoPC(P-18:1/0:0)	0.0007	0.7255	0.0126	0.5747	0.0067	0.6140
Mannitol	0.0001	0.7812	0.0002	0.7614	0.0002	0.7730
Oxoadipic acid	0.0280	0.5170	0.1511	0.3527	0.0107	0.5851
PA(8:0/a-13:0)	0.0210	0.5390	0.1023	0.3975	0.0174	0.5524
PC(14:1/20:0)	0.0069	0.6126	0.0629	0.4470	0.0382	0.4917
PC(16:0/20:2)	0.0004	0.7399	0.0027	0.6629	0.0015	0.6904
PC(18:2/16:0)	0.0009	0.7135	0.0072	0.6098	0.0019	0.6794
PC(18:3/22:6)	0.0027	0.6639	0.0142	0.5667	0.0033	0.6536
PC(20:4/18:0)	0.0540	0.4613	0.3691	0.2251	0.0794	0.4241
Prostaglandin E2	0.0016	0.6883	0.0178	0.5508	0.0008	0.7172

Name	Lactobacillus		Escherichia-Shigella		Bacillus	
	p value	|r|	p value	|r|	p value	|r|
Thymine	0.0480	0.4719	0.2465	0.2880	0.0283	0.5163

Table S7. Gut microbiota and signaling pathways associated with GH.

Name	Lactobacillus		Escherichia-Shigella		Bacillus	
	p value	|r|	p value	|r|	p value	|r|
Bax	0.0058	0.6221	0.0556	0.4586	0.0148	0.5640
Bcl-2	0.0007	0.7216	0.0044	0.6378	0.0001	0.7797
Cleaved Caspase-3	0.0058	0.6221	0.0608	0.4502	0.0148	0.5640
iNOS	0.0013	0.6967	0.0023	0.6712	0.0002	0.7631
p-p65	0.0005	0.7341	0.0011	0.7045	0.0003	0.7548
p-IκBα	0.0005	0.7341	0.0011	0.7045	0.0003	0.7548
NLRP3	0.0010	0.7092	0.0039	0.6441	0.0003	0.7548
claudin-1	0.0013	0.6967	0.0032	0.6545	0.0009	0.7133
occludin	0.0017	0.6843	0.0028	0.6608	0.0005	0.7382
ZO-1	0.0004	0.7465	0.0024	0.6691	0.0001	0.7797
Nrf2	0.0010	0.7092	0.0038	0.6462	0.0002	0.7714
Keap-1	0.0056	0.6247	0.0491	0.4699	0.0152	0.5622
p-mTOR	0.0072	0.6096	0.0556	0.4586	0.0067	0.6138
p-AMPK	0.0005	0.7341	0.0041	0.6420	0.0005	0.7382

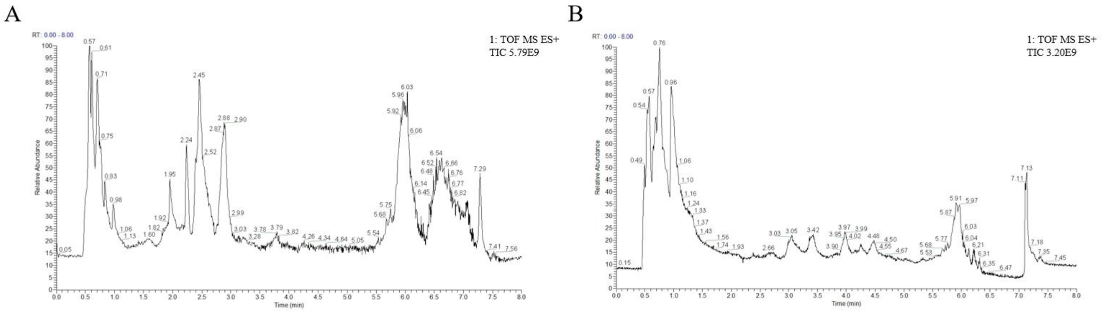

Figure S1 Total ion chromatograms (TIC) of serum. (A) Positive ion modes, (B) Negative ion modes.

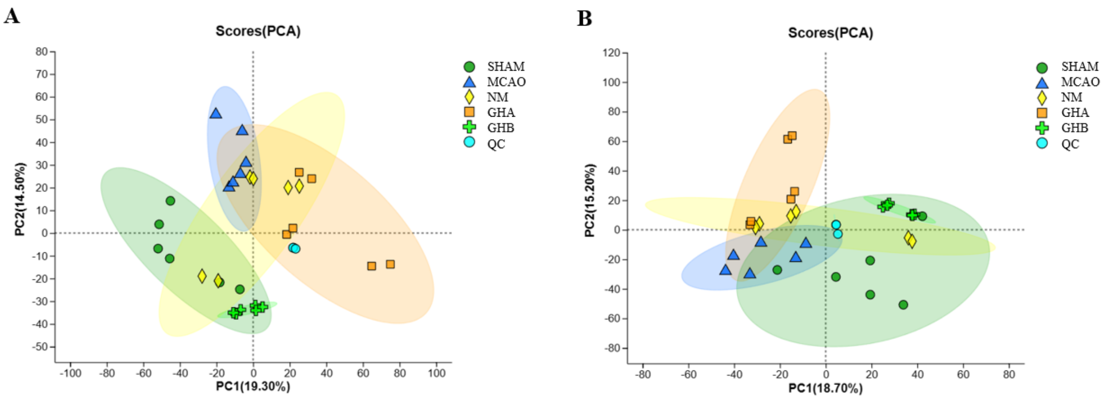

Figure S2 The PCA score plots of the five group, including ESI^+ and ESI^- ion modes.

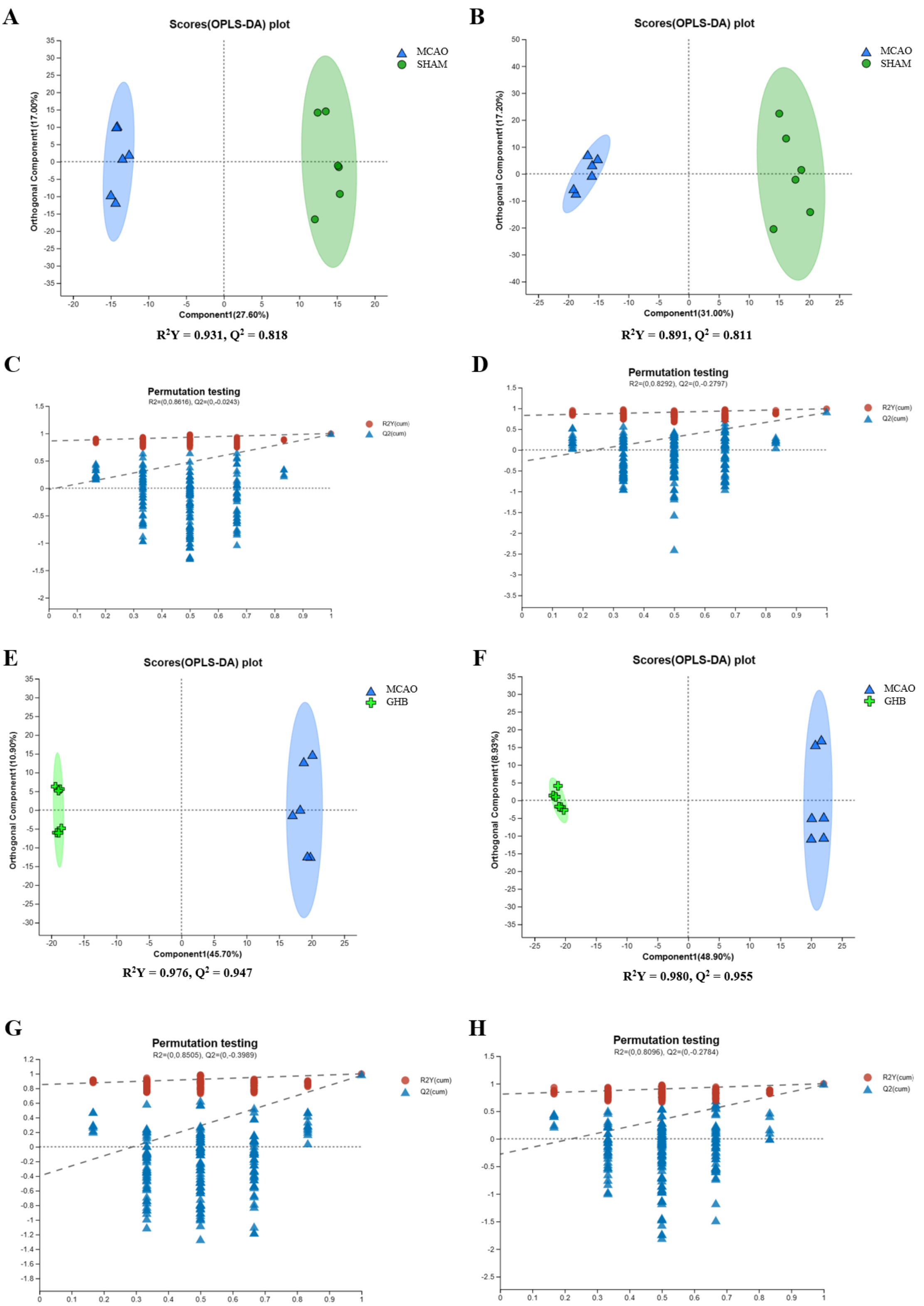

A
Scores(OPLS-DA) plot
MCAO
SHAM
Orthogonal Component1(17.00%)
Component1(27.60%)
R²Y = 0.931, Q² = 0.818
B
Scores(OPLS-DA) plot
MCAO
SHAM
Orthogonal Component1(17.20%)
Component1(31.00%)
R²Y = 0.891, Q² = 0.811
C
Permutation testing
R2=(0,0.8616), Q2=(0,-0.0243)
R2Y(cum)
Q2(cum)
D
Permutation testing
R2=(0,0.8292), Q2=(0,-0.2797)
R2Y(cum)
Q2(cum)
E
Scores(OPLS-DA) plot
MCAO
GHB
Orthogonal Component1(10.90%)
Component1(45.70%)
R²Y = 0.976, Q² = 0.947
F
Scores(OPLS-DA) plot
MCAO
GHB
Orthogonal Component1(8.93%)
Component1(48.90%)
R²Y = 0.980, Q² = 0.955
G
Permutation testing
R2=(0,0.8505), Q2=(0,-0.3989)
R2Y(cum)
Q2(cum)
H
Permutation testing
R2=(0,0.8096), Q2=(0,-0.2784)
R2Y(cum)
Q2(cum)

I **J**

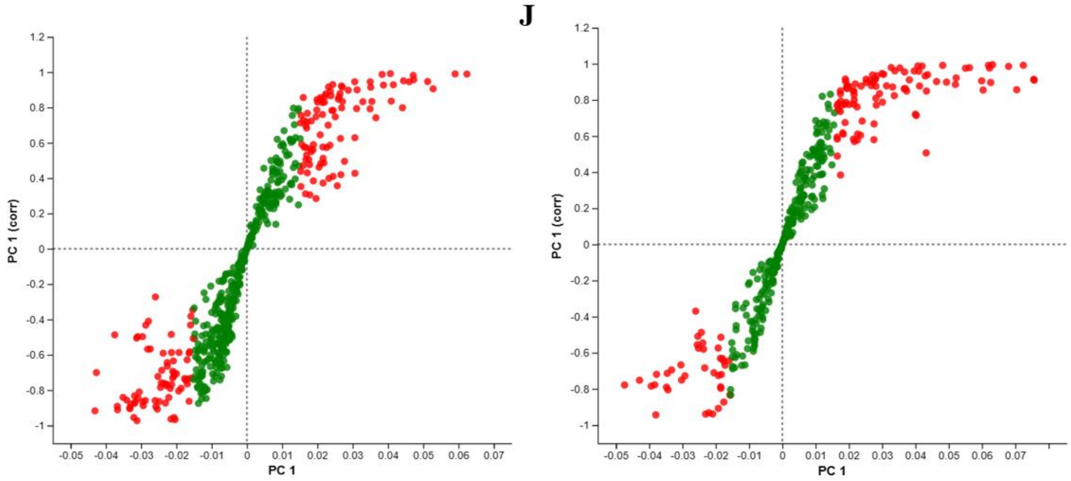

Figure S3 The OPLS-DA score plots of MCAO vs SHAM, including ESI^+ (A) and ESI^- (B) ion modes; The permutation test of MCAO vs SHAM, including ESI^+ (C) and ESI^- (D) ion modes; The OPLS-DA score plots of MCAO vs GHB, including ESI^+ (E) and ESI^- (F) ion modes; The permutation test of MCAO vs GHB, including ESI^+ (G) and ESI^- (H) ion modes. OPLS-DA S-plot of MCAO vs SHAM, including ESI^+ (I) and ESI^- (J) ion modes.

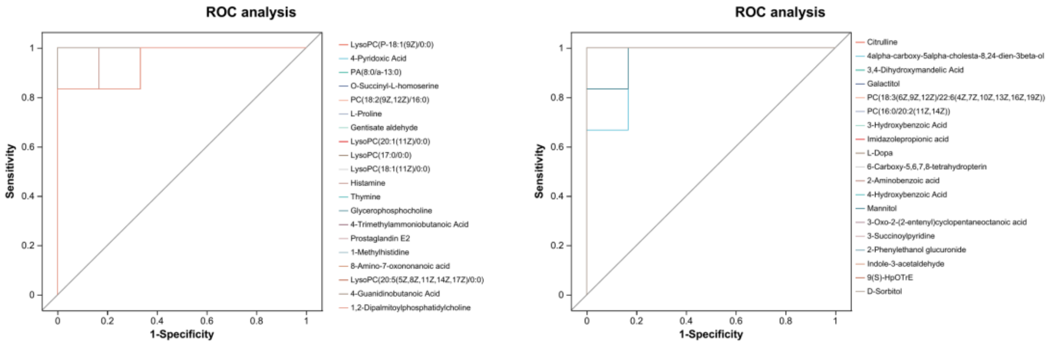

Figure S4 ROC analysis of differential metabolites.
